# Mis17 Is a Regulatory Module of the Mis6-Mal2-Sim4 Centromere Complex That Is Required for the Recruitment of CenH3/CENP-A in Fission Yeast

**DOI:** 10.1371/journal.pone.0017761

**Published:** 2011-03-21

**Authors:** Yoshiharu Shiroiwa, Takeshi Hayashi, Yohta Fujita, Alejandro Villar-Briones, Nobuyasu Ikai, Kojiro Takeda, Masahiro Ebe, Mitsuhiro Yanagida

**Affiliations:** 1 Graduate School of Biostudies, Kyoto University, Sakyo-ku, Kyoto, Japan; 2 Okinawa Institute of Science and Technology Promotion Corporation, Onna, Okinawa, Japan; University of Edinburgh, United Kingdom

## Abstract

**Background:**

The centromere is the chromosome domain on which the mitotic kinetochore forms for proper segregation. Deposition of the centromeric histone H3 (CenH3, CENP-A) is vital for the formation of centromere-specific chromatin. The Mis6-Mal2-Sim4 complex of the fission yeast *S. pombe* is required for the recruitment of CenH3 (Cnp1), but its function remains obscure.

**Methodology/Principal Findings:**

Mass spectrometry was performed on the proteins precipitated with Mis6- and Mis17-FLAG. The results together with the previously identified Sim4- and Mal2-TAP precipitated proteins indicated that the complex contains 12 subunits, Mis6, Sim4, Mal2, Mis15, Mis17, Cnl2, Fta1-4, Fta6-7, nine of which have human centromeric protein (CENP) counterparts. Domain dissection indicated that the carboxy-half of Mis17 is functional, while its amino-half is regulatory. Overproduction of the amino-half caused strong negative dominance, which led to massive chromosome missegregation and hypersensitivity to the histone deacetylase inhibitor TSA. Mis17 was hyperphosphorylated and overproduction-induced negative dominance was abolished in six kinase-deletion mutants, *ssp2* (AMPK), *ppk9* (AMPK), *ppk15* (Yak1), *ppk30* (Ark1), *wis4* (Ssk2), and *lsk1* (P-TEFb).

**Conclusions:**

Mis17 may be a regulatory module of the Mis6 complex. Negative dominance of the Mis17 fragment is exerted while the complex and CenH3 remain at the centromere, a result that differs from the mislocalization seen in the *mis17-362* mutant. The known functions of the kinases suggest an unexpected link between Mis17 and control of the cortex actin, nutrition, and signal/transcription. Possible interpretations are discussed.

## Introduction

The centromere is a region of the chromosomal DNA where duplicated sister chromatids are tightly connected. During the mitotic periods of cell division, centromeric chromatin binds to various proteins to form the kinetochore structure. The kinetochore is attached to kinetochore microtubules of the mitotic spindle in a bi-oriented fashion. The centromere DNA sequences are diverse in different organisms, but are known to bind to common proteins that form the interphase centromeric chromatin and the mitotic kinetochore.

The short centromeric DNA sequence of the budding yeast *Saccharomyces cerevisiae* contains several hundred base pairs [1], [2], while the domain centromere DNA of the fission yeast *Schizosaccharomyces pombe* is more than 100 times longer (30–100 kb) [3]. In higher eukaryotes, centromere DNA consists of further longer tandem repetitive sequences [4], [5], [6], often called satellite DNAs, of sizes on the order of megabases. Centromere/kinetochore chromatin consists of ∼100 proteins in even low eukaryotes, many of which are also present in vertebrates [7], [8], [9], [10], [11]. Thus, the basic protein composition of the centromere/kinetochore structure is largely conserved.

CENP-A is a centromere protein that associates with centromeric/kinetochoric chromatin throughout the cell cycle. Because CENP-A is centromere-specific histone H3 [12], [13], it is also called CenH3. The protein is present in all eukaryotes so far examined. CenH3/CENP-A plays crucial roles in centromeric chromatin formation and in kinetochore function, as indicated by the finding that mutations in CenH3 cause massive chromosome missegregation. In *S. pombe*, regular histone H3 is loaded in the centromeric region if Cnp1 (*S. pomb*e CenH3/CENP-A) is impaired [14].

The mechanism of CenH3 recruitment is complex and not universal among evolutionarily distant organisms. The chaperone-like proteins that directly bind to CenH3 (Scm3 in *S. cerevisiae* and *S. pombe*, HJURP in human) are remotely homologous [15], [16], [17]. Scm3-HJURP families are not found in many other organisms, including nematode and fly. In fission yeast, the recruitment of Scm3 and Cnp1/CenH3 to the centromere requires the complex of Mis18 and Mis16, both of which are present in human and are required for CENP-A recruitment [14], [18], [19], [20].

The histone acetylation-deacetylation process is crucial for the regulation of CenH3 deposition [19]. The Mis16-Mis18 complex is also required for Mis6 recruitment to the centromere. A putative homolog of Mis6 is present in the budding yeast *S. cerevisiae* Ctf3 [21], but it is not required for CenH3 loading. Instead, Ctf3 localization at the centromere requires CenH3 [22]. In vertebrates, while the depletion of CENP-I, the Mis6 homolog, was initially reported to produce no effect on CENP-A localization [23], it was later shown to be required for the deposition of newly synthesized CENP-A onto centromere [24]. Therefore, the necessity of Mis6, Ctf3, and CENP-I in CenH3/CENP-A recruitment is conserved but also variable among organisms. Furthermore, nematode and fly homologs for these proteins have not been identified. Whether the molecular functions of Mis6, Ctf3, and CENP-I have any common properties remains to be clarified.

This study focused on the function of the *S. pombe* centromere/kinetochore protein Mis17, which was originally identified as one of the proteins required for the recruitment of Cnp1/CenH3 [18]. Mis17 forms a functional group with Mis6 and Mis15, because of their mutual dependency for recruitment to the centromere/kinetochore. The central centromeric localization of Mis17 proteins throughout the cell division cycle is dependent on the co-presence of Mis6 and Mis15. Conversely, the centromeric localization of Mis6 and Mis15 requires the presence of Mis17. In *mis6*, *mis15*, and *mis17* mutants, centromeric chromatin is greatly disrupted and Cnp1/CenH3/CENP-A is not recruited to the centromere [18], [25].

Mis6 was the first of two proteins identified as minichromosome instability (*mis*) mutants, and displays the characteristic unequal mitosis [26], [27]. Mis6 acts before or at the onset of the S phase. Mitotic missegregation defects are produced once after crossing G1/S at 36°C. By contrast, the action points of Mis15 and Mis17 in the cell cycle are different [18]. For missegregation to occur, *mis15* and *mis17* mutant cells had to be continuously maintained at the non-permissive temperature from the previous mitosis. In other words, kinetochore proteins had to be maintained in an inactive state for a longer period. The restoration of protein functions at any point between two mitoses enabled cells to segregate their chromosomes correctly in the subsequent mitosis.

These results suggest that the centromere chromatin structure can be ‘remodeled’ throughout the cell division cycle. A defective centromere chromatin structure can be ‘cured’ if the correct components are provided during the cell cycle prior to the fatal mitosis. Although the molecular natures of Mis6, Mis15, and Mis17 are poorly understood, they should play important roles in the organization of centromeric chromatin throughout the cell cycle.

## Results

### Mass spectrometric analysis of the Mis6 and Mis17 complex

Mass spectrometric analysis of the *S. pombe* Mis6-Mal2-Sim4 complex was previously performed using Sim4- and Mal2-TAP [28]. We examined whether the complex was heterogeneous in subunit composition by using other subunits, i.e., Mis6- and Mis17-FLAG. Antibodies against FLAG were used to precipitate the FLAG-tagged strains whose Mis6- and Mis17-FLAG proteins were expressed by the genes integrated chromosomally under the native promoter. For comparison, a strain chromosomally expressing Mis12-FLAG integrated and expressed under the native promoter was used [19]. Mis12 is a subunit of a heterotetrameric complex functionally distinct from the Mis6 complex.

Proteins co-precipitated with Mis17-, Mis6-, or Mis12-FLAG were run on SDS-PAGE, and the gel slices were analyzed by mass spectrometry after tryptic digestion. Table 1 shows the MW (kD), number of peptides, and known orthologs in humans and budding yeast. In the previous study [28], 13 and nine proteins were co-precipitated with Sim4-TAP and Mal2-TAP, respectively. In the present study, 12 proteins (Sim4, Mal2, Mis6, Mis15, Mis17, Fta1, Fta2, Fta3, Fta4, Fta6, Fta7, and Cnl2) were commonly precipitated with Mis17-FLAG and Mis6-FLAG, but not with Mis12-FLAG. Eleven of them were previously reported among those that co-precipitated with Sim4-TAP and Mal2-TAP.

**Table 1:** Mass spectrometric analysis of *S. pombe* proteins co-precipitated with Mis17-FLAG, Mis6-FLAG, and Mis12-FLAG, and their human and *S. cerevisiae* homologs.

		Number of unique peptides			Homolog	
Protein	MW	Mis17-FLAG	Mis6-FLAG	Mis12-FLAG	Human	*S. cerevisiae*
**Mis6**	79 kD	27	35	3	CENP-I	CTF3
**Mis15**	48 kD	18	19	1	CENP-N	CHL4
**Mis17**	50 kD	25	13	2		
**Sim4**	32 kD	11	19	0	CENP-K	
**Mal2**	35 kD	26	17	0	CENP-O	MCM21
**Fta1**	32 kD	11	12	0	CENP-L	
**Fta2**	41 kD	14	14	4	CENP-P	
**Fta3**	25 kD	7	9	0	CENP-H	
**Fta4**	27 kD	13	11	1	CENP-U	
**Fta6**	7 kD	4	5	1		
**Fta7**	29 kD	24	15	1	CENP-Q	
**Cnl2**	22 kD	11	8	0		NKP2
**Sar1**	21 kD	9	7	1	Sar1a, b	SAR1
**Sam50**	52 kD	5	5	0		SAM50
**Prp19**	54 kD	4	6	1	Prp19	PRP19
**Mis12**	30 kD	1	1	19	hMis12	MTW1
**Ndc80**	72 kD	0	2	35	Hec1/hNdc80	NDC80
**Dad1**	10 kD	1	7	2		DAD1

We did not detect Fta5 in the precipitates of the Mis6- or Mis17-FLAG strain. The Mis6-recruited centromeric protein, Cnl2 [29] was a common component of the complex. Dad1 [28] was present in the Mis6-FLAG co-precipitates but was scarcely seen in the Mis17-FLAG precipitates. Consistently, Liu et al. [28] did not detect Mis17 in Dad1-coprecipitated proteins. These previous results, combined with those of the present study, suggest that the Mis6 complex consists of these 12 common subunits.

Sar1, Sam50, and Prp19 were present in the immunoprecipitates with both Mis6- and Mis17-FLAG, but not with Mis12-FLAG. As Sar1, Sam50, and Prp19 do not localize with the centromere/kinetochore [30], the significance of their co-precipitation with Mis6 and Mis17 remains to be determined. As a control, proteins that precipitated with Mis12-FLAG were analyzed. They contained each of the four subunits of the Ndc80 and the Mis12 complex besides Spc7, none of which were present in the Mis6- and Mis17-containing complex. Hence, *S. pombe* Mis12, Ndc80, and Spc7 form a supra-molecule that is distinct from the Mis6 complex in *S. pombe*.

The budding yeast *S. cerevisiae* Ctf19 complex that contains Ctf19, Mcm21, Okp1, Mcm22, Mcm16, Ctf3, Chl4, Mcm19, Nkp1, Nkp2, Ame1, and Mtw1 [31] might be the counterpart complex, because the amino acid sequences of Mal2, Mis6, Mis15, and Cnl2 are similar to those of Mcm21, Ctf3, Chl4, and Nkp2, respectively. However, the *S. pombe* complex did not contain Mis12 (Mtw1 homolog) as described here. In vertebrates, a similar complex may also exist, because nine subunits, Mal2, Fta1, Fta2, Fta4, Fta7, Mis6, Mis15, Fta3 and Sim4, are similar to CENP-O, CENP-L, CENP-P, CENP-U, CENP-Q, CENP-I, CENP-N, CENP-H, and CENP-K, respectively (Table 1) [32]. Judging from the amino acid sequences, only three gene families, Mis6-Ctf3-CENP-I, Mis15-Chl4-CENP-N, and Mal2-Mcm21-CENP-O, are conserved across fission yeast, budding yeast, and human.

While Mis17 was suggested to be similar to budding yeast Ctf19 [32], the similarity is not certain. The coiled coil of Mis17 is found in the C-terminus, while that of Ctf19 exists in the N-terminus. The budding yeast Ctf19 is a subunit of the heterotetrameric subcomplex COMA (Ctf19, Okp1, Mcm21, Ame1) [33]. However, a counterpart for COMA has not been found in *S. pombe*.

### Mis17 is highly phosphorylated

Mass spectrometric analysis suggested that Mis17 might contain phosphorylated sites. Three possible sites, RIS^94^E, VSS^105^I, and ENS^166^PP, were found in an analysis that covered 66% of the 441-amino acid (aa) sequence. Phosphorylation at these sites has not been confirmed. Note that these sites are located in the N-terminal region of Mis17.

**Figure 1:**
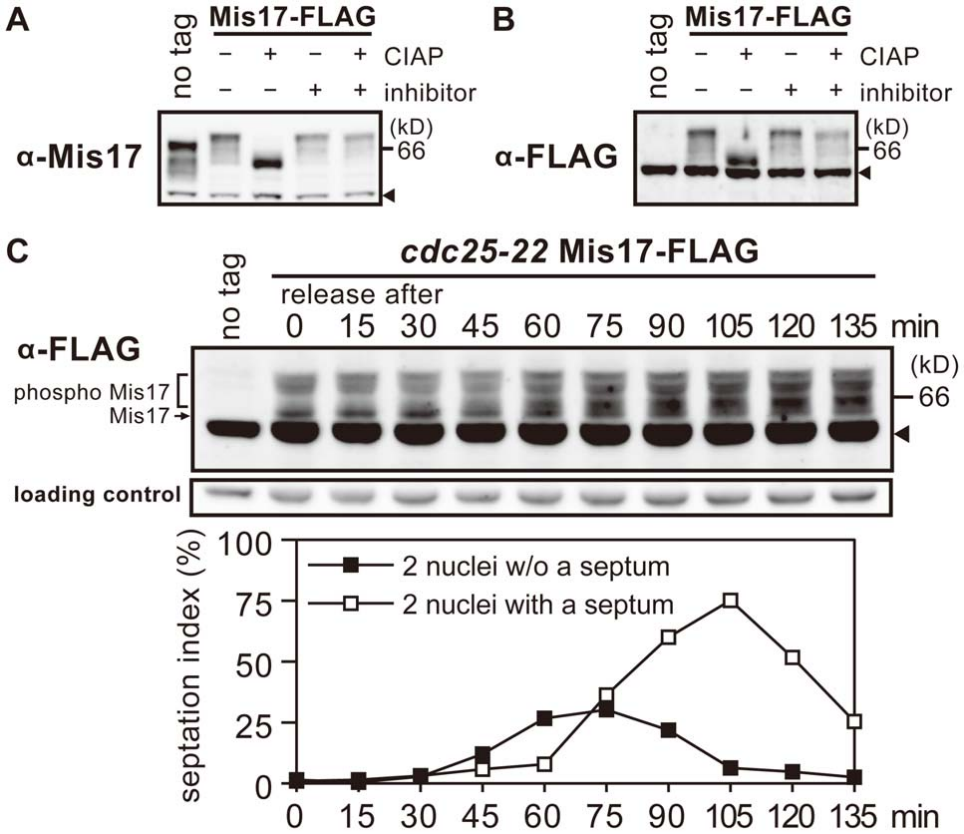
Mis17 is a hyperphosphoprotein. **A–B**. An SDS-PAGE of *S. pombe* cell extracts was performed to detect Mis17 and Mis17-FLAG by immunoblot, using antibodies against bacterial-made Mis17 (α-Mis17 in **A**) and FLAG (α-FLAG in **B**). The wild-type strains with no tag and the chromosomally-integrated Mis17-FLAG gene expressed under the native promoter were used. Cell extracts of the Mis17-FLAG strain were treated with the calf intestine alkaline phosphatase (CIAP) in the presence (+) or absence (−) of phosphatase inhibitors (β-glycerophosphate and p-nitrophenyl phosphate). Arrowheads indicate the cross-reacting antigens. The no-tag band migrated faster than the Mis17-FLAG band with the expected MW difference. **C**. Mis17-FLAG chromosomally integrated in the *cdc25-22* mutant and expressed under the native promoter was detected by immunoblot at 15 min intervals from 0–135 min (top). Arrowhead indicates the cross-reacting antigens. A shorter exposed image of the cross-reacting antigens is shown as the loading control (middle). The mutant cells were arrested in the G2 phase at 36°C, and released to mitosis by shifting the temperature to 26°C. Cells synchronously progressed with the timing of chromosome segregation, septation, and cytokinesis at 45, 75, and 105 min, respectively (bottom). Frequencies of cells showing two nuclei without (filled squares) or with (open squares) a septum were measured. See text for changes of the Mis17-FLAG band in the synchronous culture.

To detect the Mis17 protein by immunoblot, we made bacterially-produced GST-Mis17 and obtained rabbit polyclonal antibodies, which were used after affinity purification. To tag Mis17 with FLAG, the wild-type *mis17^+^* gene was tagged with FLAG, chromosomally integrated, and expressed under the native promoter. As shown in Figure 1A and B, both native Mis17 and tagged Mis17-FLAG were detected in *S. pombe* extracts by immunoblot using antibodies against Mis17 or FLAG (α-Mis17 and α-FLAG, respectively). The no-tag intact Mis17 was not detected by the anti-FLAG antibody. Cross-reacting antigens in the extracts are indicated by arrowheads in the figures. The bands of intact Mis17 and tagged Mis17-FLAG were broad and multiple. They were more heavily concentrated at the bottom position after treatment with phosphatase CIAP, but not after treatment with phosphatase inhibitors, β-glycerophosphate and p-nitrophenyl phosphate. Mis17 was thus hyperphosphorylated. Mis17 sequence contains many phosphorylatable residues (69 S, 21 T, and eight Y).

### The phosphorylation pattern of Mis17 may partly change during the cell cycle

A *cdc25* mutant block-release experiment was performed to examine whether the phosphorylation bands of Mis17 change during mitosis. The *cdc25* mutant cells [34] were synchronously arrested in the G2 phase at the restrictive temperature (36°C) for 4 h, and then released into mitosis upon shifting to the permissive temperature (26°C). As shown in Figure 1C, the band of Mis17 in the G2-arrested cells seemed to be hypophosphorylated compared with that of the asynchronously growing cells. Upon the release of arrested cells into mitosis by the shift from 36 to 26°C, phosphorylation of this band increased around 60 min (i.e., chromosomal segregation) and again around 105 min (i.e., cell separation/cytokinesis). After exiting the G2 phase, Mis17 might become more phosphorylated in the metaphase-anaphase transition, and then further phosphorylated prior to cytokinesis in the G1 and S phases (i.e., when the newly synthesized centromeric protein is loaded). Mis17 thus seems to be highly phosphorylated throughout the cell cycle, and additional phosphorylation and dephosphorylation might occur from mitosis through the G1/S phase.

### Distinct overproduction effects of the C-terminal and N-terminal fragments

The above results suggest that Mis17 might be a target of post-translational regulation in the Mis6 complex (MW ∼430,000). To make a functional dissection of Mis17, we constructed plasmids carrying various parts of Mis17. The carboxy- and amino-terminal halves of Mis17 showed distinct phenotypes.

**Figure 2:**
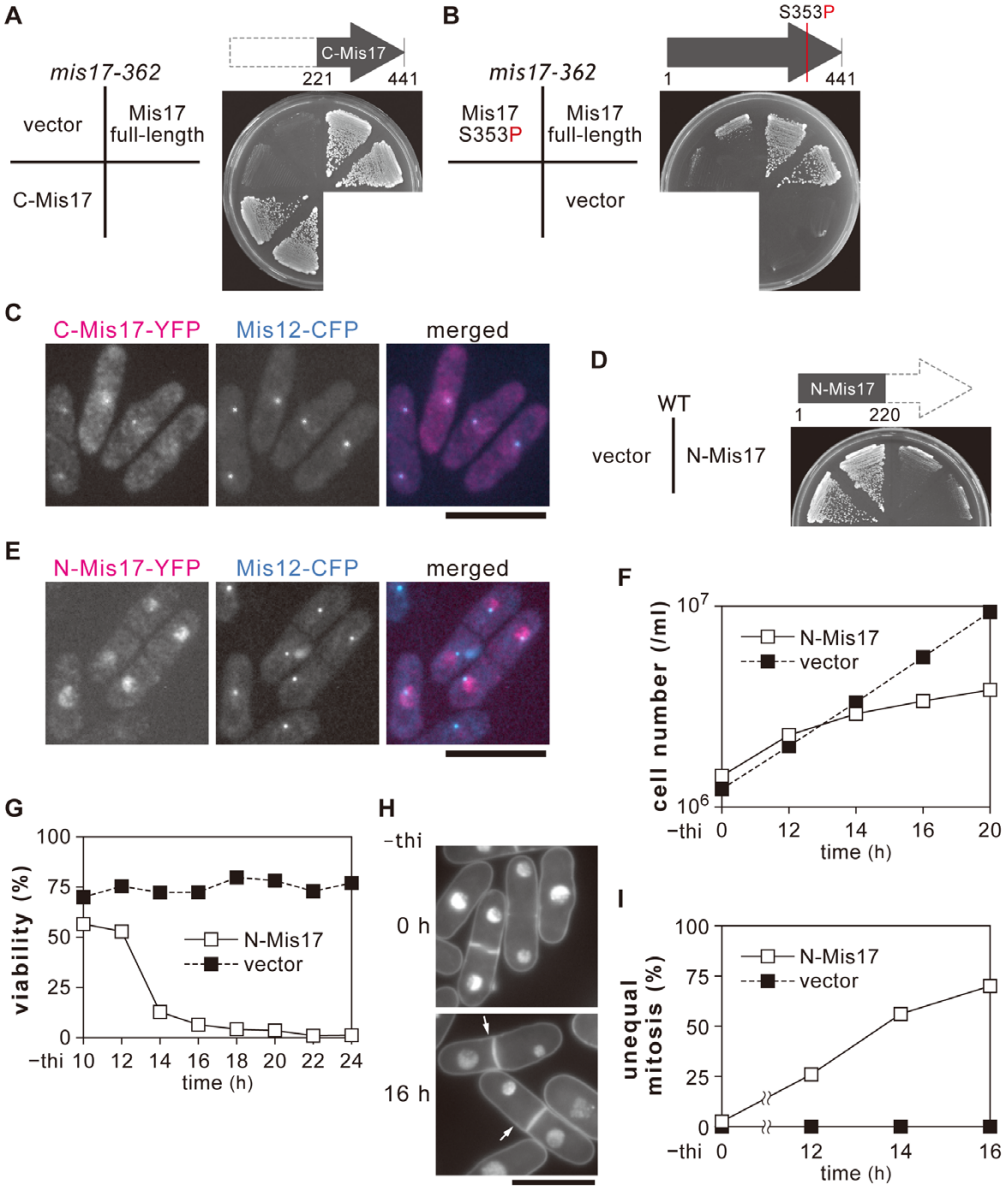
Overproduction of N-Mis17 is toxic. **A–C**. Plasmid REP41 carrying the wild-type C-Mis17 gene (the carboxy-terminal half of Mis17) under the *nmt1* promoter was made and introduced into the *mis17-362* mutant. When the C-Mis17 was moderately expressed in the presence of thiamine, the plasmid rescued the ts phenotype (**A**). Plasmid carrying Mis17 with the substitution mutation (Mis17 S353P) failed to rescue *mis17-362* (**B**). The mutant was not rescued by the control vector, but was rescued by plasmid carrying the wild-type full-length Mis17 gene. To visualize the localization of C-Mis17, plasmid carrying the C-Mis17-YFP was introduced into a strain that was also chromosomally integrated with Mis12, an authentic centromere/kinetochore gene that had been tagged with CFP and expressed under the native promoter. C-Mis17-YFP was overexpressed at 26°C for 16 h in the absence of thiamine (the mildest level of overexpression by the promoter *nmt*1 REP81). The signals of C-Mis17-YFP were localized at the centromere/kinetochore dots (**C**, Bar, 10 µm). **D**. N-Mis17 plasmid (REP41) inhibited colony formation of the wild-type strain in the absence of thiamine. **E**. N-Mis17-YFP in the plasmid REP81 was expressed in the strain that also carried Mis12-CFP, and observed 16 h after overexpression in the absence of thiamine at 26°C. Bar, 10 µm. **F-G**. The cell number of the strain carrying plasmid N-Mis17 (REP41 promoter) was counted after overexpression in the absence of thiamine (−thi) at 33°C (**F**). The cell viability was measured by plating the wild-type carrying vector or REP41 plasmid carrying N-Mis17 (**G**). After 16 h in the absence of thiamine, the majority of wild-type cells carrying N-Mis17 had lost viability. **H–I**. DAPI-stained *S. pombe* wild-type cells carrying the plasmid N-Mis17 fragment (REP41 promoter) after 16 h in the absence of thiamine at 33°C (**H**, Bar, 10 µm). Arrows indicate cells showing the large and small daughter nuclei typical for defects in centromere-binding proteins. Quantitative data for the frequency of chromosome missegregation were obtained by counting the number of unequal-mitotic cells in binuclear ones (**I**).

The carboxy-terminal half (aa 221–440, designated C-Mis17) was moderately expressed in the ts mutant *mis17-362*. As shown in Figure 2A, *mis17-362* mutant cells were fully rescued by a plasmid that overexpressed C-Mis17, forming colonies at 36°C. The vector served as the negative control, and the plasmid pMIS17 carrying the full-length mis17^+^ gene served as the positive control. C-Mis17 was therefore functional like full-length Mis17 in the high-copy plasmid. Plasmid carrying the full-length *mutant* gene Mis17-S353P failed to rescue the ts phenotype (Figure 2B).

To visualize the localization of C-Mis17, YFP-tagged C-Mis17 was expressed in the absence of thiamine (promoter, on) under the promoter *nmt1* using the plasmid REP81 (the mildest promoter), in a strain that carried the chromosomally integrated Mis12-CFP (an authentic centromere marker) under the native promoter. C-Mis17-YFP expression was somewhat diffuse, perhaps due to overproduction. C-Mis17-YFP existed as centromeric/kinetochoric dots (Figure 2C), consistent with the ability of C-Mis17 to rescue the ts phenotype of *mis17-362*.

Overproduction of the amino-terminal half of Mis17 (aa 1–220; designated N-Mis17) caused the opposite effect of that seen with C-Mis17 (Figure 2D). The wild-type cells barely formed colonies when N-Mis17 was overproduced by the plasmid REP41 at 33°C in the absence of thiamine (normal colonies were made in the presence of thiamine, as the promoter is off). N-Mis17-YFP was not located on the centromere, but was broadly present in the nuclear chromatin when expressed in the absence of thiamine (Figure 2E). When N-Mis17 was overproduced by REP41, the cell number increase was diminished (Figure 2F) and the cell viability sharply declined (Figure 2G) after 14 h. DAPI-stained micrographs revealed a high frequency of unequal chromosome segregation (arrows in Figure 2H), consistent with the quantitative rise in unequal segregation (Figure 2I). This phenotype of unequal chromosome segregation is typical for the *mis6* and *mis17* mutants [18], [27]. Thus, N-Mis17 was lethal to the wild-type cells because overproduction caused missegregation.

### N-Mis17 causes swift missegregation under the TET promoter

**Figure 3:**
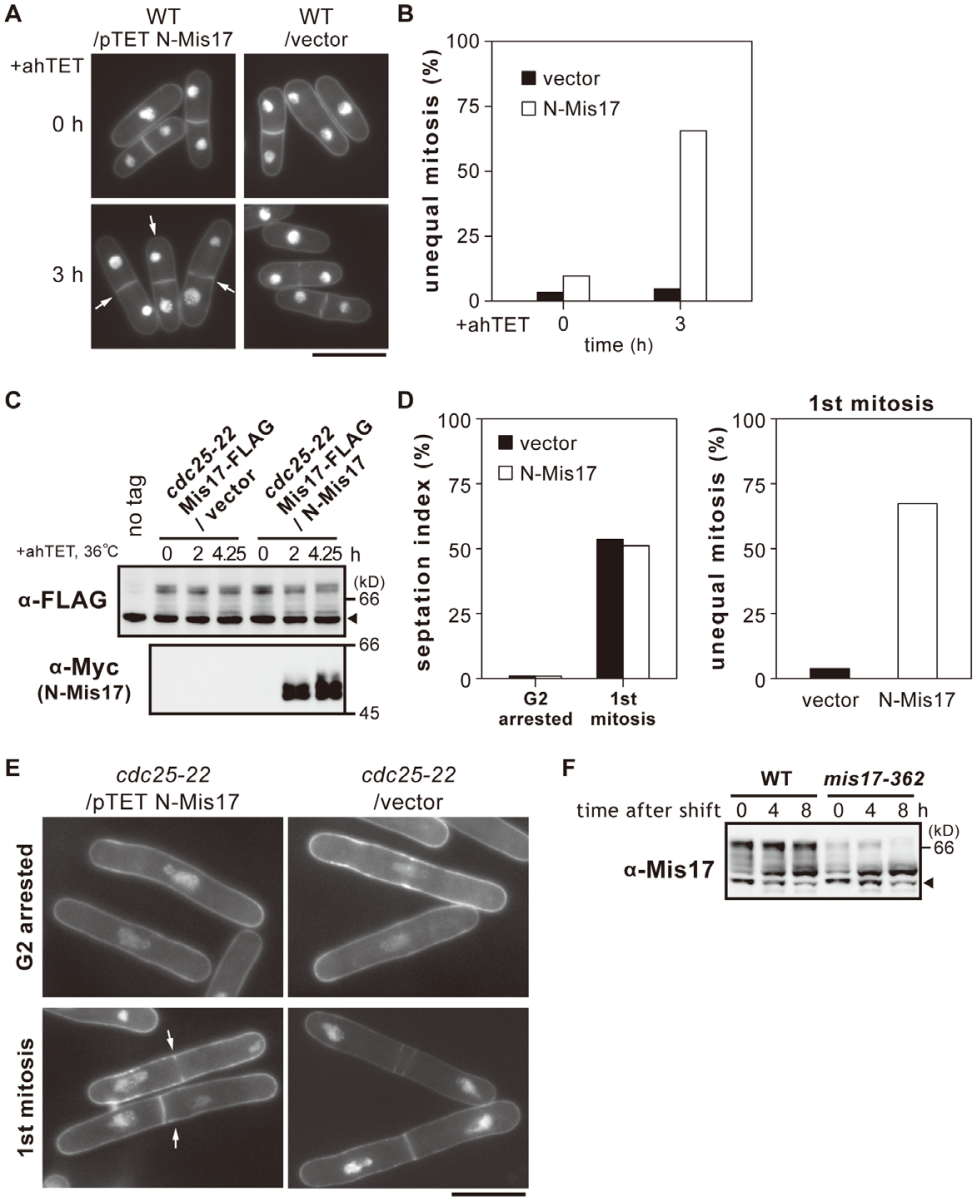
Rapid occurrence of missegregation by N-Mis17 under the TET promoter. **A**. Chromosome missegregation was frequently observed after TET-induced overproduction of N-Mis17 in wild-type cells at 33°C as shown in the DAPI-stained micrograph (arrows). Bar, 10 µm. **B**. N-Mis17 was expressed under the TET promoter. The frequencies of chromosome segregation (%) were measured at 0 and 3 h after induction by the anhydrotetracycline (ahTET) at 33°C. **C–E**. The *cdc25-22* mutant was transformed by plasmid carrying N-Mis17-Myc expressed under the TET promoter. Endogenous Mis17-FLAG was chromosomally integrated and expressed under the native promoter. Mis17-FLAG and N-Mis17-Myc were assayed by immunoblotting using antibodies against FLAG and Myc. Arrowhead indicates the cross-reacting antigens. The *cdc25-22* mutant cells that expressed endogenous Mis17-FLAG and ectopically produced N-Mis17-Myc were arrested after 4.25 h at 36°C. See text for explanation of (**C**). After the release of mutant cells to 26°C, the frequencies of chromosome missegregation and the septation index were measured (**D**). Micrograph of missegregation (arrows) is shown in (**E**, Bar, 10 µm). **F**. Immunoblotting was done to detect Mis17 protein in the extracts of the wild-type and ts mutant *mis17-362* cells at 36°C for 0–8 h. The *mis17-362* mutant protein was unstable. Arrowhead indicates the cross-reacting antigens.

The *nmt1* promoter-dependent overproduction required a long time (12–15 h) before expression. To shorten the time for induction, we applied the TET promoter that should be inducible within a short time period (1–2 h). N-Mis17 was overproduced in the wild-type strain by plasmid carrying the N-Mis17 under the TET promoter (Materials and Methods). The frequency of unequal chromosome segregation massively increased upon overproduction (arrows in Figure 3A): 3 h after induction, 65% of the cells displayed unequal mitosis (Figure 3B). The time required for the appearance of a dominant negative effect in mitosis was thus greatly reduced compared with that of the *nmt1* promoter.

We next examined whether chromosome missegregation could occur in mitotic cells that had accumulated N-Mis17 in the preceding interphase. The *cdc25-22* strain that contained the chromosomally integrated Mis17-FLAG was transformed with a plasmid carrying Myc-tagged N-Mis17 (N-Mis17-Myc) under the TET promoter. The vector plasmid was used as a control. The strain was cultured at 36°C for 4.25 h, such that the *cdc25* mutant cells were blocked at the G2 phase. The TET promoter was induced at the same time that the temperature was increased. As shown in Figure 3C, the phosphorylation band patterns of the endogenous Mis17-FLAG remained nearly constant after 4.25 h. By contrast, N-Mis17-Myc was already massively produced after 2 h at 36°C and had accumulated in the G2-arrested *cdc25-22* cells. Note that the bands of Mis17 were hyper-phosphorylated in the synthetic medium EMM2 employed here, while those in the rich YPD medium were relatively hypo-phosphorylated particularly when cells were blocked in the late G2 phase in *cdc25* mutant cells, thus producing more bands (see Figure 1C).

The culture was then released to 26°C, and synchronous cells were examined at the time of septum formation (75 min after release). The septation index reached ∼50% (Figure 3D, left panel). The frequency of chromosomal missegregation was 67% in cells carrying the plasmid N-Mis17-Myc, but only 3.8% in cells carrying the vector (right panel). DAPI-stained micrographs of cells displaying missegregation after 75 min in the released culture are shown in Figure 3E (arrows). These results demonstrate that N-Mis17 caused missegregation in the first mitosis after its induced accumulation in interphase.

### The mutant ts *mis17-362* protein is deficient in the quantity

Affinity-purified polyclonal antibodies (α-Mis17) were used to detect the mutant Mis17 protein in the *mis17-362* strain, which was cultured at 36°C for 8 h. The mutant protein band was diminished even at the permissive temperature of 26°C (0 h, Figure 3F). In the C-terminal coiled-coil of *mis17-362*, the S^353^ residue is altered to P, a helix-breaking residue [18]. The mutant protein might be unstable due to an impaired conformation in an essential helix. Unequal mitosis was frequently observed 8 h after the temperature shift to 36°C (Figure S1).

We then constructed the chromosomally integrated GFP-tagged mutant *mis17-362* gene, which displayed diminished GFP signals at 36°C (Figure S2). Consistent with the diminished protein level of *Mis17-362*, the GFP signal intensity of Mis17^ts^-GFP was low even at 26°C. The phenotypes produced by *mis17-362* were thus due to a loss of functional Mis17 protein at the centromere, in contrast to the gain of negative dominance by N-Mis17.

### N-Mis17 overproduction does not affect endogenous Mis17

**Figure 4:**
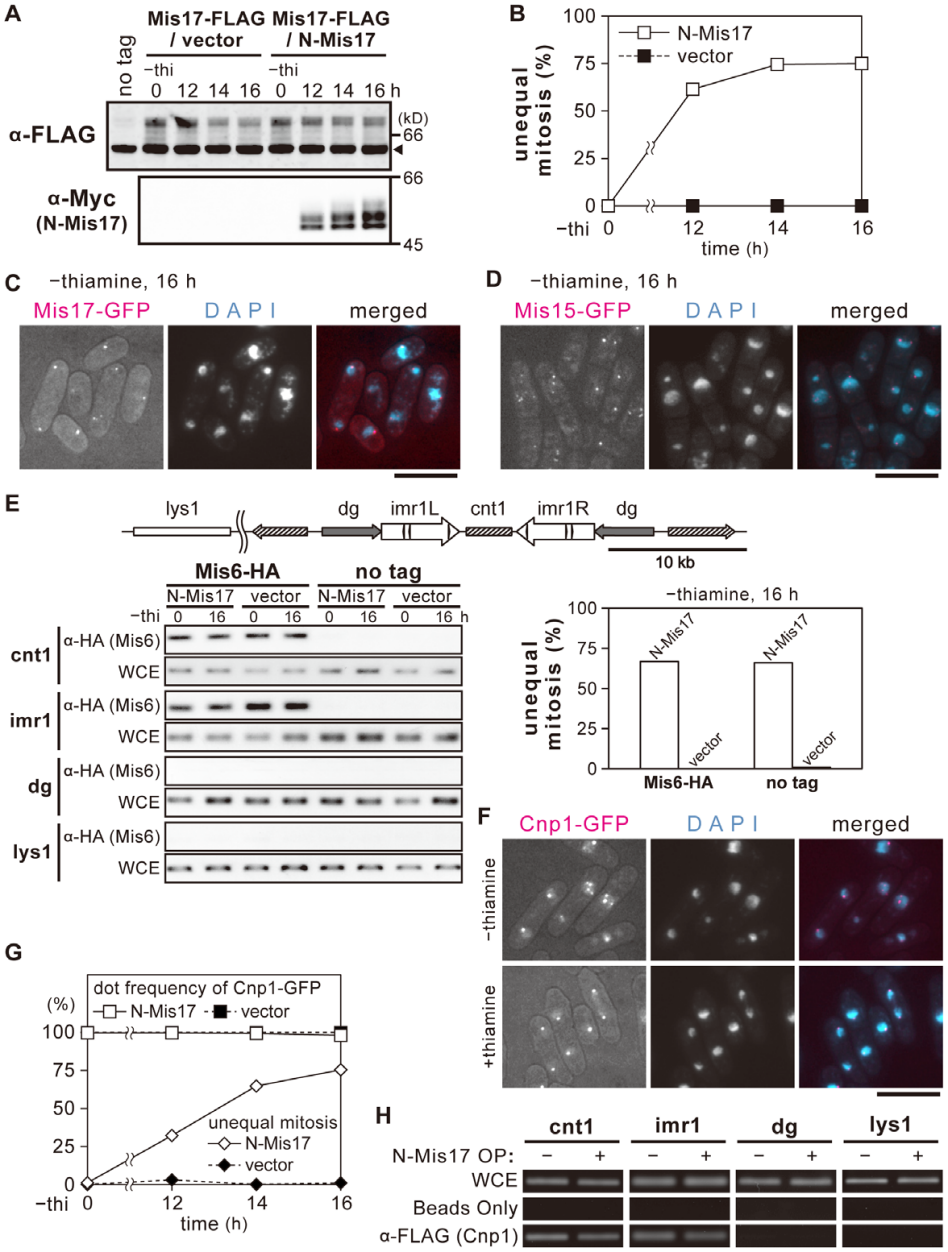
N-Mis17 overproduction does not affect endogenous centromere proteins. **A**. Vector plasmid or the inducible REP41 plasmid carrying the N-Mis17-Myc gene was introduced into a strain in which endogenous Mis17-FLAG was chromosomally integrated and expressed under the native promoter. N-Mis17-Myc and Mis17-FLAG were assayed by immunoblot. The phosphorylation bands of endogenous Mis17 is shown in the upper panel (α-FLAG). Those of overproduced N-Mis17-Myc is in the lower panel (α-Myc). Arrowhead indicates the cross-reacting antigens. **B**. The frequency of missegregation was measured after shifting to the absence of thiamine. **C**. Endogenous Mis17-GFP was observed in cells that overproduced the N-Mis17 fragment in the absence of thiamine (16 h at 33°C). Bar, 10 µm. **D**. Mis15-GFP was observed in cells that overproduced N-Mis17 in the absence of thiamine (16 h at 33°C). Bar, 10 µm. **E**. Chromatin immunoprecipitation (ChIP) was done to examine whether Mis6 was bound to the central centromere in cells that overproduced N-Mis17 at 33°C. The central centromere DNAs, such as cnt1 and imr1, were precipitated with Mis6 (α-HA) in the wild-type cells that overproduced N-Mis17 (left panel). Chromosome missegregation was most frequent while Mis6 remained at the central centromere (right panel). **F**. Cnp1/CenH3 was observed in cells that overproduced N-Mis17 at 33°C. Cnp1-GFP chromosomally integrated and expressed under the native promoter was observed in the absence (top, overproduction for 16 h) and presence (bottom, overproduction is off) of thiamine. Bar, 10 µm. **G**. The frequencies of Cnp1-GFP signals at the centromere/kinetochore and the frequencies of chromosome missegregation were quantified. **H**. ChIP was performed for the strain that was integrated with Cnp1/CenH3-FLAG under the native promoter and transformed with plasmid carrying N-Mis17. When N-Mis17 was overproduced (16 h at 33°C), Cnp1/CenH3 was bound to the central centromeric cnt1 and imr1.

The state of endogenous Mis17 was examined when N-Mis17 was overproduced under the *nmt1* REP41 promoter. No recognizable changes were observed in the quantity or degree of phosphorylation of endogenous Mis17-FLAG (without thiamine, promoter on), while the level of N-Mis17-Myc was greatly increased at 12–16 h (Figure 4A). The frequency of chromosome missegregation was maximal around 14–16 h after thiamine removal (Figure 4B). In cells carrying the control vector plasmid, the frequency of chromosome segregation was negligible. Taken together, overproduction of N-Mis17 under *nmt1* and TET did not interfere with endogenous Mis17 at the protein level or with the phosphorylation band patterns.

We examined whether the localization of endogenous Mis17-GFP was affected in cells that overproduced N-Mis17. As shown in Figure 4C, endogenous Mis17 (chromosomally tagged with GFP and expressed under the native promoter) appeared to be associated with the dot-like centromere/kinetochore under overproduction, while the mitotic chromosomes were extensively missegregated. Quantitative data for the centromere/kinetochore localization and the degree of missegregation are shown in Figure S3. Nearly all cells contained Mis17-GFP signals at the centromere/kinetochore, and the frequency of missegregation was >70%. Furthermore, the centromere/kinetochore localization of Mis15-GFP (chromosomally integrated and expressed under the native promoter) was apparently normal in cells that overproduced N-Mis17-GFP, while chromosome missegregation was extensive (Figure 4D and Figure S4).

### N-Mis17 does not dislocate Mis6 or CenH3 from the central centromere

Chromatin immunoprecipitation (ChIP) was performed to examine whether Mis6 and CenH3/Cnp1 were localized at the centromere/kinetochore in wild-type cells that overproduced N-Mis17. As shown in Figure 4E (left panel), Mis6 tagged with HA (Mis6-HA) and integrated onto chromosome under the native promoter was normally precipitated by anti-HA antibody with the central centromeric DNA (cnt1 and imr1) but not with pericentric dg DNA or non-centromeric lys1 DNA before (0 h) and after (16 h) overproduction. The result was quite similar to that of non-overproduction control. The no-tag control is also shown. The frequency of chromosome missegregation estimated by DAPI-staining was 67% (right panel). Thus, Mis6 was found in the central core of the centromere, while missegregation extensively took place through the overproduction of N-Mis17.

We next asked whether GFP-tagged Cnp1/CenH3, chromosomally integrated and expressed under the native promoter, also remained at the centromere/kinetochore when N-Mis17 was overproduced. As shown in Figure 4F and G, the Cnp1-GFP dot signals were still present but de-clustered at 16 h, when missegregation was most frequent. We also examined Cnp1-GFP expression in the presence of thiamine (overproduction is off), and found that the Cnp1-GFP signals clustered normally.

To confirm this light microscopic observation, ChIP was performed after N-Mis17 was overproduced. Cnp1-FLAG was co-precipitated with the central centromeric DNAs, cnt1 and imr1, but not with the pericentric repetitive dg sequence or the lys1 unique gene, similar to Mis6. This finding indicates that both Cnp1/CenH3 and Mis6 were positioned normally at the central centromeric region in the presence of overproduced N-Mis17 (Figure 4H). Taken together, these results indicate that Mis17, Mis15, Mis6, and Cnp1 remained normally bound to the centromere/kinetochore.

### N-Mis17 overproducing cells are sensitive to HDAC inhibitor

**Figure 5:**
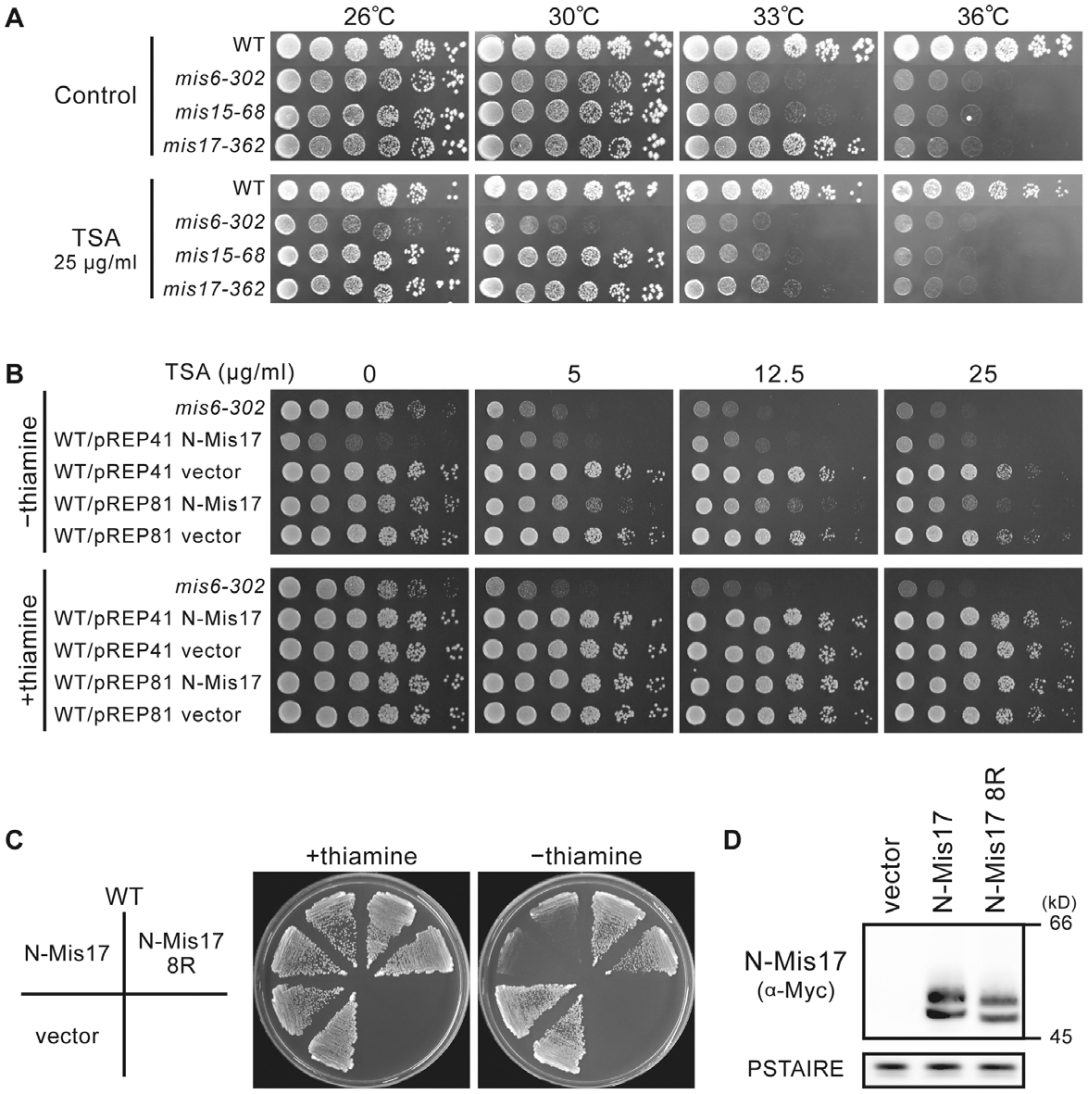
N-Mis17 overproduction causes sensitivity to TSA. **A**. Wild type, *mis6-302*, *mis15-68*, and *mis17-362* were spotted on a plate in the absence or presence of 25 µg/ml TSA at 26–36°C. See text. **B**. Wild-type cells that overproduced N-Mis17 by REP41 or REP81 were plated in the absence or presence of TSA (0–25 µg/ml) at 30°C. **C**. The N-Mis17 fragment contains eight K residues that were substituted to R. The resulting N-Mis17-8R gene was expressed under the REP41 promoter in the wild-type strain. **D**. The level of overproduced Mis17-N 8R was assayed compared with N-Mis17. PSTAIRE was the level of Cdc2 protein kinase assayed by the antibody PSTAIRE.

Mis6 is reportedly sensitive to the histone deacetylase (HDAC) inhibitor trichostatin A (TSA) [35]. Therefore, we examined whether *mis17-362* was also hypersensitive to TSA. As shown in Figure 5A, *mis17-362* failed to produce colonies at 33°C, a semi-permissive temperature, in the presence of 25 µg/ml TSA, while the wild-type formed normal colonies under the same condition. The TSA sensitivity of the *mis15-68* mutant was approximately equal to that of *mis17-362*, but the *mis6-303* mutant cells were sensitive to TSA even at the permissive temperature (26°C). Wild-type cells producing N-Mis17 under the *nmt1* promoter (using the REP81 plasmid) were sensitive to 5–25 µg/ml TSA at 30°C, while wild-type cells carrying the vector were not (Figure 5B).

We next tested whether substitution mutants in N-Mis17 affect the negative dominance of the N-Mis17 fragment. Because there are a very large number of S and T residues in N-Mis17, making an S and T substitution mutant difficult, R substitutions were instead made for the eight K residues in N-Mis17. These residues might be acetylated, methylated, or ubiquitinated. As shown in Figure 5C, the 8-R mutant (designated Mis17-N 8R) completely lost the negative dominance effect when it was overproduced. The level of overproduced Mis17-N 8R was comparatively lower (∼2-fold) than that of the wild type N-Mis17 (Figure 5D). The 8R form might be migrated faster as it is more basic than the wild type 8K form, and might become unstable, resulting into the reduction of the Mis17-8R level, which might explain the rescue effect.

### Negative dominance is backed by actin- and nutrition-related kinases

**Figure 6:**
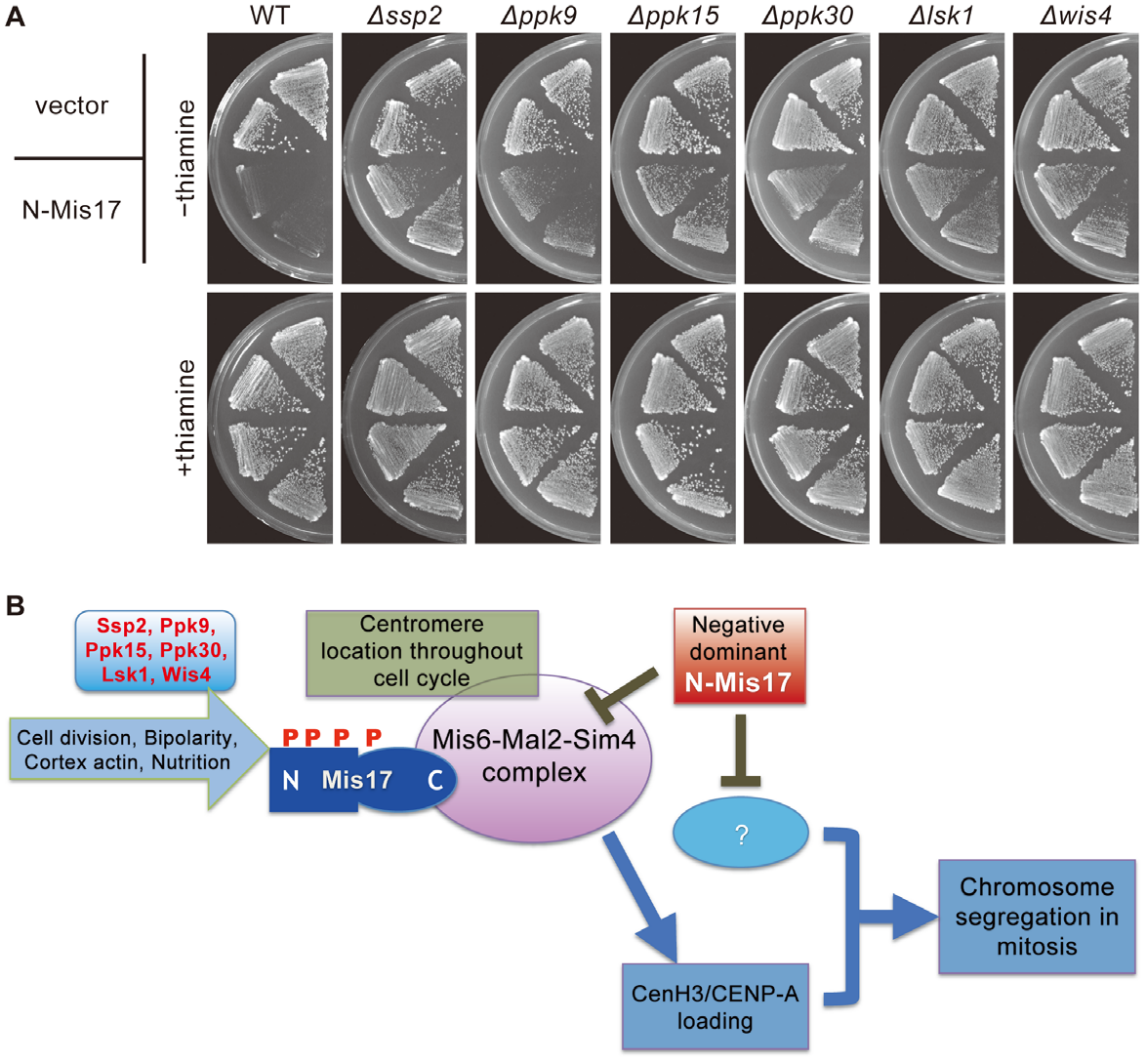
Negative-dominance is supported by certain protein kinases. **A**. The negative dominance effect of N-Mis17 overexpression was greatly diminished in six protein kinase deletion mutants, Δ*ssp2*, Δ*ppk9*, Δ*ppk15*, Δ*ppk30*, Δ*lsk1*, and Δ*wis4.* Promoter is off or on in the presence or absence of thiamine, respectively. **B**. A cartoon for the implication of Mis17 in the Mis6-Mal2-Sim4 complex and negative dominance effect of the N-Mis17 fragment. See text.

We considered the possibility that certain kinases might affect the negative dominance effect of N-Mis17, and employed a set of kinase deletions [36] that were transformed by the REP41 plasmid responsible for N-Mis17 overproduction. Fifty-five transformed strains were plated in the presence or absence of thiamine. The negative dominance effect of N-Mis17 was virtually abolished in six deletion mutants, Δ*ssp2*, Δ*ppk9*, Δ*ppk15*, Δ*ppk30*, Δ*lsk1*, and Δ*wis4* (Figure 6A).

Unexpectedly, none of the deletion mutants are related to centromere/kinetochore function, but rather are related to the cortical actin cytoskeleton, cell bipolarity, and/or glucose availability. Ssp2 and Ppk9 are catalytic subunits of Snf1-like AMP-PK [37] and are implicated in the consumption of glucose energy [38]. Each of Ssp2 and Ppk9 binds to two regulatory proteins (Spcc1919.03c designated AMPB and Cbs2) that are similar to the mammalian β- and γ-subunits of AMPK [37]. Ppk30 resembles the budding yeast Ark1/Prk1 family kinase that helps to regulate the cortical actin cytoskeleton and endocytosis [39], [40]. Lsk1 resembles a P-TEFb-associated cyclin-dependent kinase that is responsible for phosphorylation of the RNA polymerase II CTD domain [41], [42]. Lsk1 also resembles a latrunculin-sensitive kinase implicated in the maintenance of the contractile ring at the cell equator [43]. Ppk15, Ssp2, and Wis4 positively regulate the establishment of bipolar cell growth [44]. Ppk15 is also similar to the budding yeast Yak1 kinase that is part of the glucose-sensing system involved in growth control in response to glucose availability [45] and related to actin regulation [46], [47], [48], [49]. Wis4 is similar to the budding yeast Ssk2 kinase, is a stress-activated MAPKKK, and regulates the actin cytoskeleton [50].

## Discussion

Mis17 may be a regulatory module of the Mis6 complex. Nine subunits in the Mis6 complex have human homologues as constitutive centromere proteins (CENPs; Table 1). The identification of the functions of these subunits should help elucidate the dynamics of centromeric chromatin, on which the mitotic kinetochore is made every cell cycle. The roles of these subunits are unclear except for their implication in the deposition of CenH3 in *S. pombe*. Mis17 is hyperphosphorylated, and its phosphorylation might be a key to understanding the module function of Mis17 in the Mis6 complex. However, the human complex may play a distinct role, and the budding yeast may not have the counterpart complex. Further work is necessary to understand the evolutionary conserved and non-conserved functions of the complex.

A possible common role in different organisms might be the regulation of protein deacetylation-acetylation cycle in the centromeric chromatin, as *mis6*, *mis15*, *mis17* mutations and overproduction of N-Mis17 all caused the hypersensitivity of TSA. Although *mis17-36*2 was mildly sensitive to TSA, N-Mis17 overproduction caused severe sensitivity comparable to that of the *mis6-302* mutant [35]. It may be that the Mis6 complex is an upregulator of HDAC(s) at the centromere and is inhibited by N-Mis17. Alternatively, N-Mis17 might be acetylated and functionally deplete the HDACs. Consistent with the latter possibility, K-to-R substitutions in the N-Mis17 fragment abolished the negative dominance effect of N-Mis17 overproduction. Note, however, that methylation and ubiquitination may occur at K residues, and further study is necessary to clarify these possibilities.

The level of endogenous Mis17 was not affected by overproduction of the N-Mis17 fragment. Nevertheless, massive chromosome missegregation was produced by the negative dominance effect of fragment expression. Both the slow and the fast inducible promoters used for N-Mis17 overproduction (*nmt1* and TET, respectively) showed similar phenotypes. Therefore, the duration of culture was irrelevant. The TET promoter produced maximal missegregation within 3 h after induction, indicating that missegregation likely occurs in the first mitosis after the onset of overproduction. Surprisingly, Cnp1/CenH3, endogenous Mis17, Mis6, and Mis15 were all apparently localized normally at the centromere/kinetochore during interphase and mitosis. Their protein levels were also normal. The ChIP results indicated that Cnp1/CenH3 was also located normally at the central centromere. This finding is distinct from the mislocalization or loss phenotype found in ts *mis17-362* cells [18]. It may be that the broad localization of N-Mis17 in the nuclear chromatin directly or indirectly downregulates Mis6 complex that is bound to the centromere (depicted in Figure 6B). Alternatively, the N-Mis17 fragment may inhibit an unidentified target (indicated by a question mark) essential for chromosome segregation. The unidentified target might not be implicated in CENP-A loading, but involved closely in mitotic chromosome segregation such as the kinetochore components.

The results of the block-and-release experiment using the *cdc25* mutant strain suggested that Mis17 might be constitutively phosphorylated throughout the cell cycle, with additional phosphorylation sites that can be altered during the cell division cycle. A few upper bands appeared to be intensified at the time of septum formation and cell separation, namely during G1/S when newly synthesized Cnp1/CenH3 was deposited at the centromere.

We were unable to identify any relevant phosphorylation sites in Mis17 or any protein kinases that directly phosphorylate Mis17. A clue for identifying such kinases was obtained by using a set of kinase deletion mutants [36]. Deletion of six protein kinases virtually abolished the negative dominance effect of N-Mis17 overproduction. These kinases might not directly phosphorylate Mis17, because the hyperphosphorylation bands remained in the deletion mutants (unpublished result). Multiple phosphorylation might be essential for producing negative dominance. Alternatively, the target protein(s) of N-Mis17 might be altered and escape from the negative dominance in these kinase deletions.

These six kinases are all related to interphase cell growth and cyctokinesis, and none of them are directly involved in mitosis. Five (Ppk30, Ppk15, Lsk1, Wis4, Ssp2) of the six are related to actin (cortical patch, contractile ring maintenance, and bipolar cell growth) [39], [40], [43], [44], [50], [51]. Three are required for glucose availability (Ssp2, Ppk9, Ppk15) [37], [45]. One (Lks1) is a latrunculin (actin inhibitor)-sensitive protein [43] involved in RNA polymerase II CTD regulation [41], [42], which implicates the cell cycle-dependent oscillation of many transcripts [52]. These findings suggest that Mis17 and the Mis6 complex might be involved in cortical actin and/or nutritional regulation.

One hypothesis is that the Mis6 complex functions in G1/S progression, regulating and/or recruiting the newly synthesized CenH3 and other constitutive centromeric proteins (e.g., the 12 subunits of the Mis6 complex). Since the missegregation occurs in mitosis, Mis17 might be hyperphosphorylated in interphase and further modulated during mitosis. The mitotic modulation could be targeted by the N-Mis17 fragment. Alternatively, N-Mis17 could target the mitotic component unrelated to the Mis6 complex. This hypothesized modulation of the mitotic complex could require the multiple phosphorylations observed in the preceding G1/S phase. More specifically, mitotic modulation might occur on the basis of the hyperphosphorylation that had occurred during G1/S progression. Chicken CENP-H and human CENP-U were shown to interact with Hec1/Ndc80, a kinetochore component [53], [54]. Dad1 that directly interacts with microtubule coprecipitated with the subunits of the *S. pombe* Mis6 complex as shown in Liu et al. [28] and the present study. Hence the implication of the Mis6 complex with the mitotic kinetochore components and microtubule seems to be plausible in *S. pombe*.

Then, why the ts *mis17-362* mutant is defective in CenH3 recruitment? A possible scenario is that the Mis6-Mal2-Sim4 complex might be impaired or even dissociated after a few rounds of division of *mis17-362* at 36°C, and mutant Mis17-362 protein might become hypo-phosphorylated and failed to bind to centromere, resulting the defect to recruit CenH3. Previously we showed that Mis6 and Mis15 localization at centromere was defective in *mis17-362* [18]. Another question is that could phosphorylation of the N-Mis17 half domain directly affect CenH3 recruitment? We assume that phosphorylation of N-Mis17 occurred after the Mis6 complex is made, and phosphorylation might not be directly related to CenH3 recruitment. Instead phosphorylation might be involved in the interaction with the mitotic target. N-Mis17 overproduction might compete with the mitotic function of endogenous Mis17.

## Materials and Methods

### Strains and culture media

*S. pombe* strains were derived from the haploid wild-type *972* (*h^−^*) and *975* (*h* ^+^) (see Table S1). Complete YPD and minimal EMM2 media were used [55]. Transformation was performed using the lithium method [56].

### Plasmids and gene handling

The truncated *mis17* ^+^ fragments were cloned into pREP41 or pREP81, in which expression is under the control of the weakened *nmt1* promoter [57]. The *nmt1* promoters are repressible by thiamine [58]. To chromosomally integrate tagged genes into the native loci on the genomic DNA, pYC6, pYC11 [59], and the hygromycin resistance gene containing plasmid were used. Plasmid pTET was constructed as follows. The *nmt1* promoter was excised from the pREP41 vector. Next, the PCR-amplified DNA fragment from pDUAL-tet-redβ [60], which contains the tetracycline-inducible CaMV35S promoter and the repressor gene *tetR* under the control of the *adh1* promoter, was subcloned into the vector. As an inducer of the CaMV35S promoter, 15 µM anhydrotetracycline (ahTET) was used [60].

### Microscopy

DAPI staining was performed as described [61]. To observe cells that expressed the tagged fluorescent protein, cells were adhered to a glass funnel filter and fixed by immersion in 100% methanol at −80°C. After 30 min of the methanol fixation, the cells were washed with phosphate buffered saline (PBS) until the methanol concentration reached 30%.

### Western blotting and ChIP

Protein extracts were prepared by the TCA precipitation method [62]. The ChIP method was performed as described [27]. For immunochemical methods, anti-Mis17 (described below), anti-FLAG (M2, SIGMA), anti-Myc (ab-1, oncogene), and anti-HA (12CA5, Roche) antibodies were used.

### Anti-Mis17 antibody

Anti-Mis17 polyclonal antibody was made by PEPTIDE INSTITUTE, INC. using GST-Mis17 fusion protein as antigen. The antibody was affinity purified using a membrane on which recombinant His-Mis17 was blotted.

### Alkaline phosphatase treatment

Calf intestine alkaline phosphatase (CIAP, Takara) was used for phosphatase treatment. Cells were grown in YPD at 26°C to log phase. Cell extracts were prepared with phosphatase reaction buffer (50 mM Tris-HCl [pH 9.0], 1 mM MgCl_2_, 1 mM PMSF, 1 mM DTT, and 1% Triton X-100). Lysate (90 µg) was incubated with 40 U of phosphatase for 1 h at 37°C. As phosphatase inhibitors, 60 mM β-glycerophosphate and 15 mM p-nitrophenyl phosphate were used.

### Mass spectrometry

The procedures were performed essentially as previously described [63], [64]. For immunoprecipitation, cell extracts were prepared with the extraction buffer (25 mM HEPES-KOH [pH 7.5], 200 mM NaCl, 10% glycerol, 0.1% NP-40, 1 mM PMSF). Immunopurified samples were separated on a 4–12% gradient SDS-PAGE gel. The area from the top to the bottom of the separation gel was cut at ∼1–2-mm intervals. After in-gel digestion with modified trypsin, the resulting peptides were analyzed by LC/MS/MS. To identify high-scoring proteins and phosphorylation sites of the Mis17 protein, all MSMS spectra were searched against the *S. pombe* non-redundant protein database, including common contaminants such as trypsin and keratin, with the Mascot program (Matrix Science, London, UK).

## Supporting Information

Figure S1 **Observation of unequal mitosis in the** ***mis17-362*** **mutant.** In the *mis17-362* strain, unequal mitosis was frequently observed 8 h after the temperature shift to 36°C as shown in the DAPI-stained micrograph (left panel, arrows) and the frequency measurement (right panel, filled circle). Bar, 10 µm.
(TIF)

Figure S2 **Diminishment of Mis17^ts^-GFP at the restrictive temperature.** Diminishment of GFP signals were observed in the strain with the chromosomally- integrated and GFP-tagged mutant *mis17-362* gene (Mis17^ts^-GFP) at 36°C. Bar, 10 µm.
(TIF)

Figure S3 **Localization of endogenous Mis17-GFP under the N-Mis17 overproducing-condition.** The frequencies of endogenous Mis17-GFP signals at the centromere/kinetochore and chromosome missegregation were quantified under the N-Mis17 overproducing-condition in the absence of thiamine (−thi) at 33°C.
(TIF)

Figure S4 **Localization of Mis15-GFP under the N-Mis17 overproducing-condition.** The frequencies of chromosomally integrated Mis15-GFP signals at the centromere/kinetochore and chromosome missegregation were quantified under the N-Mis17 overproducing-condition in the absence of thiamine (−thi) at 33°C.
(TIF)

Table S1 **Strains used in this study.**
(DOC)
