# Supplementary figures and images for: Mis17 Is a Regulatory Module of the Mis6-Mal2-Sim4 Centromere Complex That Is Required for the Recruitment of CenH3/CENP-A in Fission Yeast
Source: PLoS One. 2011 Mar 21;6(3):e17761. doi: 10.1371/journal.pone.0017761 (PMC3061866; doi:10.1371/journal.pone.0017761)

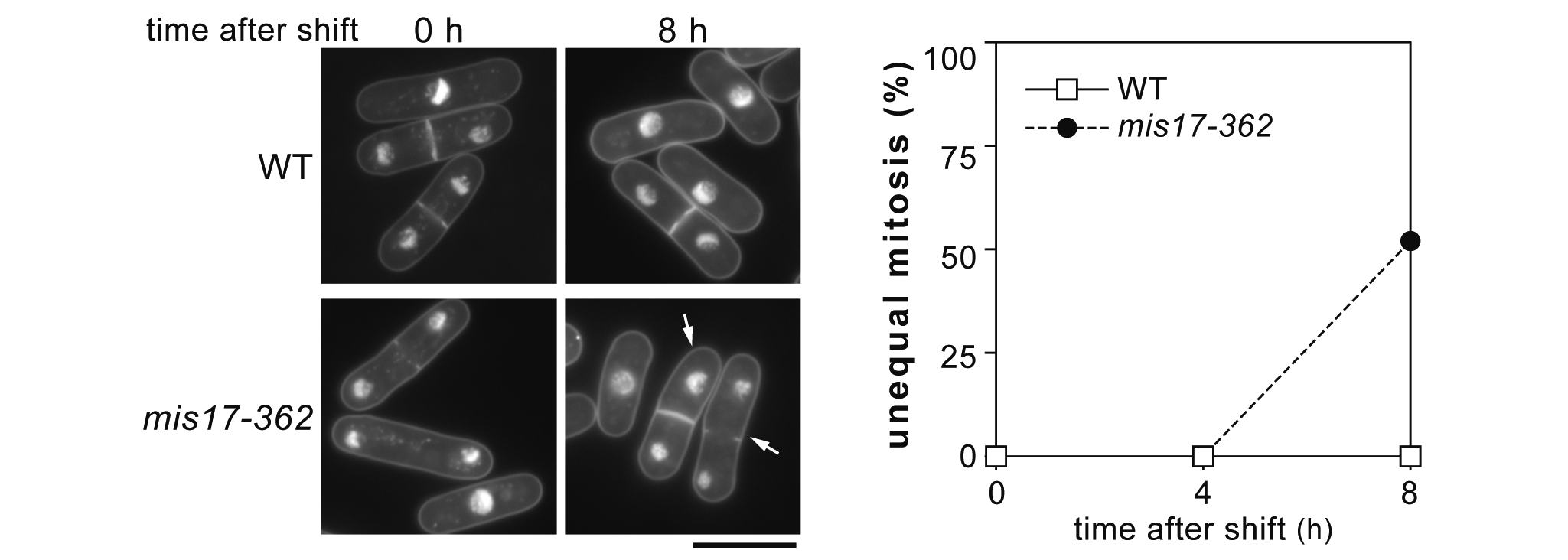

Supplement: Figure S1 — Observation of unequal mitosis in the mis17-362 mutant. In the mis17-362 strain, unequal mitosis was frequently observed 8 h after the temperature shift to 36°C as shown in the DAPI-stained micrograph (left panel, arrows) and the frequency measurement (right panel, filled circle). Bar, 10 µm. (TIF) [file pone.0017761.s001.tif]

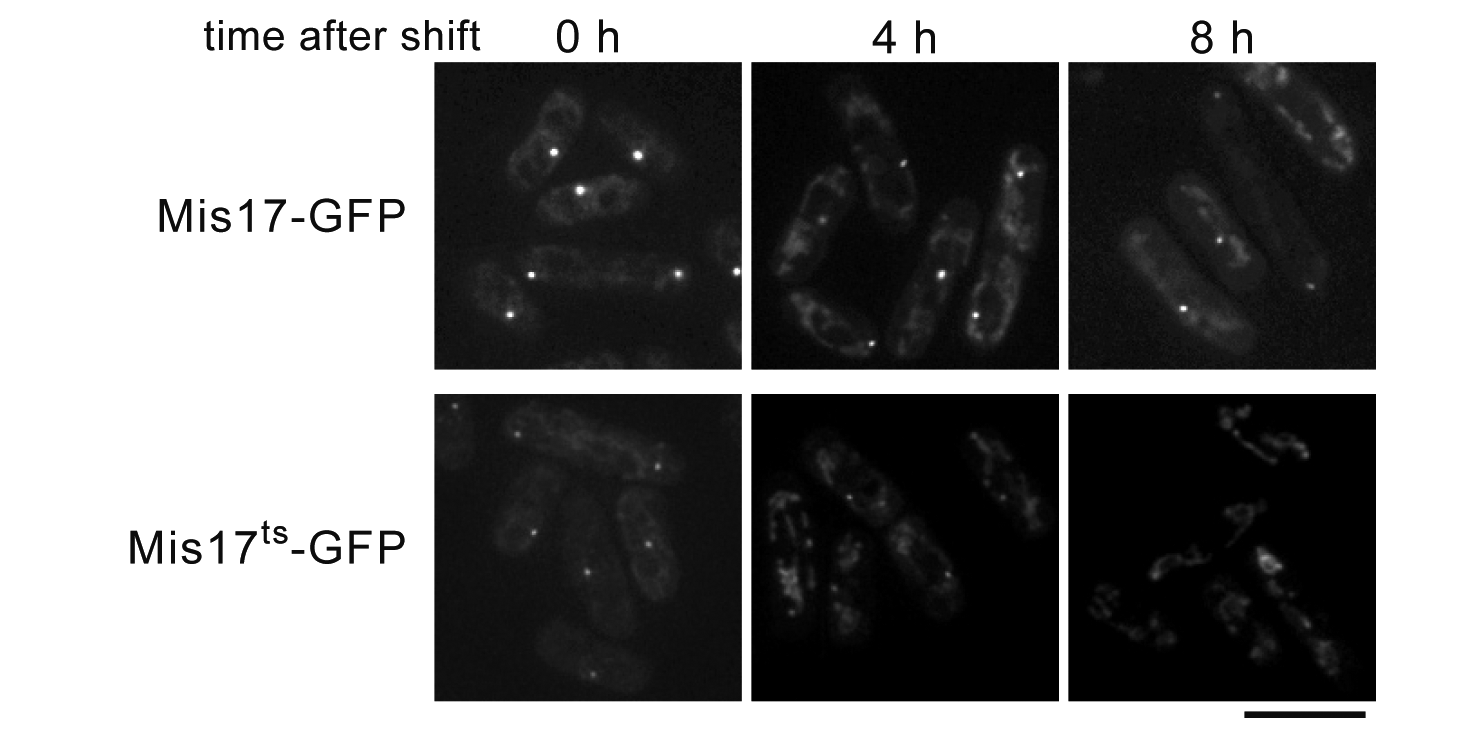

Supplement: Figure S2 — Diminishment of Mis17ts-GFP at the restrictive temperature. Diminishment of GFP signals were observed in the strain with the chromosomally- integrated and GFP-tagged mutant mis17-362 gene (Mis17ts-GFP) at 36°C. Bar, 10 µm. (TIF) [file pone.0017761.s002.tif]

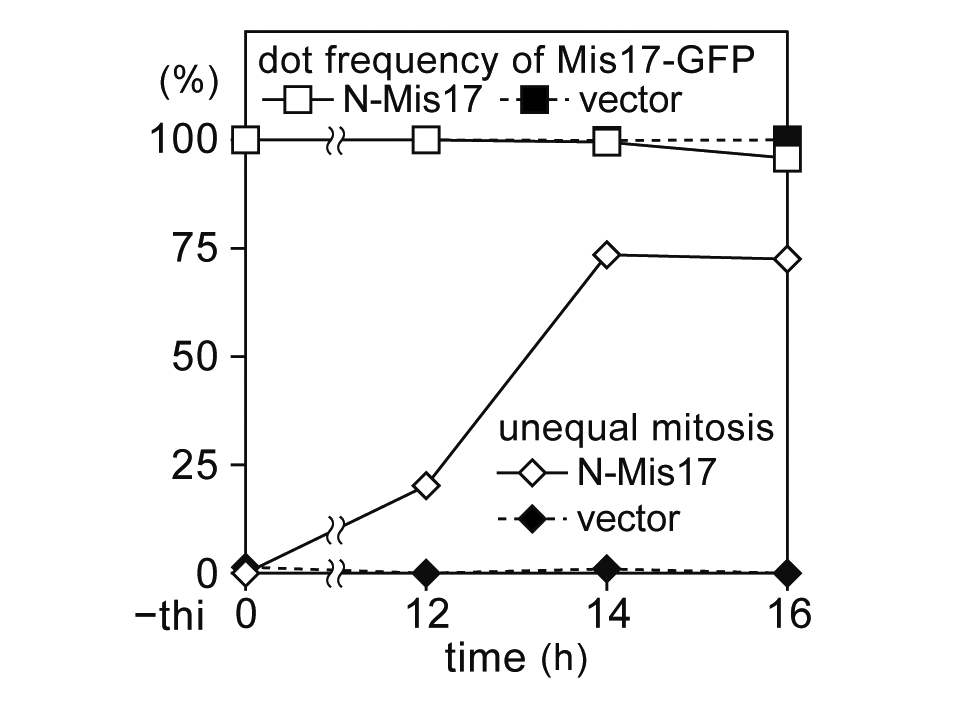

Supplement: Figure S3 — Localization of endogenous Mis17-GFP under the N-Mis17 overproducing-condition. The frequencies of endogenous Mis17-GFP signals at the centromere/kinetochore and chromosome missegregation were quantified under the N-Mis17 overproducing-condition in the absence of thiamine (−thi) at 33°C. (TIF) [file pone.0017761.s003.tif]

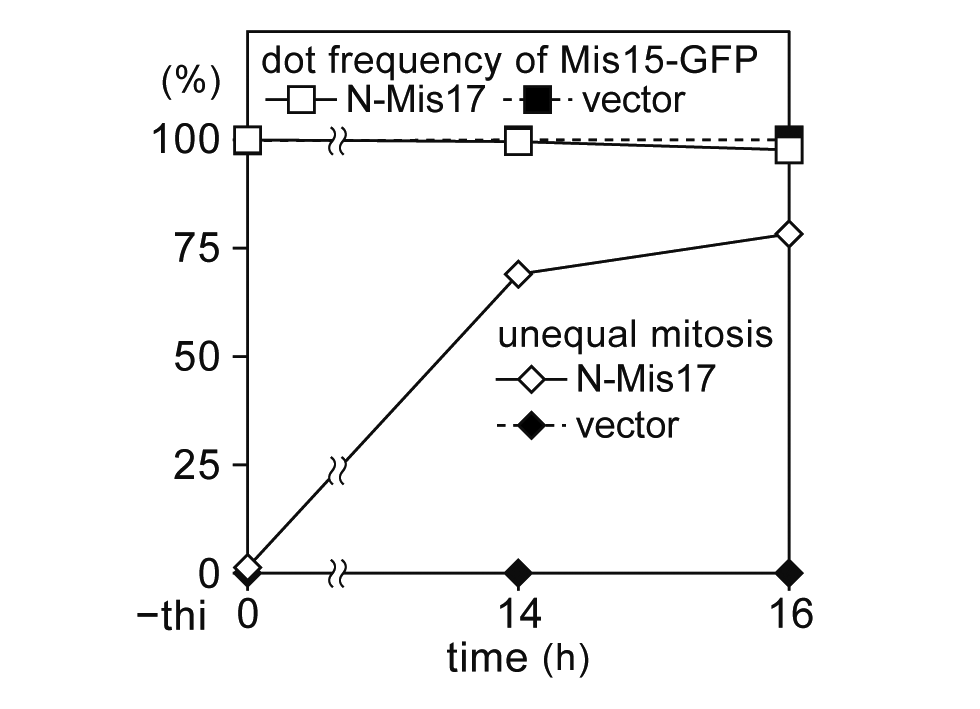

Supplement: Figure S4 — Localization of Mis15-GFP under the N-Mis17 overproducing-condition. The frequencies of chromosomally integrated Mis15-GFP signals at the centromere/kinetochore and chromosome missegregation were quantified under the N-Mis17 overproducing-condition in the absence of thiamine (−thi) at 33°C. (TIF) [file pone.0017761.s004.tif]
