# Supplementary material for: Mis17 Is a Regulatory Module of the Mis6-Mal2-Sim4 Centromere Complex That Is Required for the Recruitment of CenH3/CENP-A in Fission Yeast
Source: PLoS One. 2011 Mar 21;6(3):e17761. doi: 10.1371/journal.pone.0017761 (PMC3061866; doi:10.1371/journal.pone.0017761)
Supplement: Table S1 — Strains used in this study. (DOC) [file pone.0017761.s005.doc]

| **Genotype** | **Source** |
| --- | --- |
| *h^-^* | lab stock |
| *h^-^ leu1* | lab stock |
| *h^-^* Mis6-FLAG[KanR] | this study |
| *h^-^ leu1 ura4* Mis12-FLAG[*ura4^+^*] | Fujita et al., 2007 |
| *h^-^* Mis17-FLAG[hph] | this study |
| *h^+^ leu1* Mis17-FLAG[hph] | this study |
| *leu1* Cnp1-FLAG[KanR] | this study |
| *h^-^ cdc25-22* Mis17-FLAG[hph] | this study |
| *h^+^ leu1 cdc25-22* Mis17-FLAG[hph] | this study |
| *h^+^ mis6-302* | Takahashi et al., 1994 |
| *h^-^ mis15-68* | Hayashi et al., 2004 |
| *h^-^ mis17-362* | Hayashi et al., 2004 |
| *h^-^ leu1 mis17-362* | Hayashi et al., 2004 |
| *h^-^ leu1 ura4* Mis6-HA[*ura4^+^*] | Saitoh et al., 1997 |
| *h^-^ leu1 ura4* Mis12-CFP[*ura4^+^*] | Obuse et al., 2004 |
| *h^-^ leu1 ura4* Mis15-GFP[*ura4^+^*] | Hayashi et al., 2004 |
| *h^-^ leu1 ura4* Mis17-GFP[*ura4^+^*] | this study |
| *h^-^ leu1* Mis17^ts^-GFP[Leu2*^+^*] | this study |
| *h^-^ leu1 lys1^+^*::Cnp1-GFP | Takahashi et al., 2000 |
| *h^-^ leu1 ura4 ssp2Δ*::*ura4 ^+^* | Bimbó et al., 2005 |
| *h^-^ leu1 ura4 ppk9Δ*::*ura4 ^+^* | Bimbó et al., 2005 |
| *h^-^ leu1 ura4 ppk15Δ*::*ura4 ^+^* | Bimbó et al., 2005 |
| *h^-^ leu1 ura4 ppk30Δ*::*ura4 ^+^* | Bimbó et al., 2005 |
| *h^-^ leu1 ura4 lsk1Δ*::*ura4 ^+^* | Bimbó et al., 2005 |
| *h^-^ leu1 ura4 wis4Δ*::*ura4 ^+^* | Bimbó et al., 2005 |
